# Conversion to mTOR-Inhibitors Plus IV Immunoglobulins in Kidney-Transplant Recipients with BKV Infection: A Retrospective Comparative Study

**DOI:** 10.3390/jcm11247292

**Published:** 2022-12-08

**Authors:** Carla Vela, Thomas Jouve, Eloi Chevallier, Farida Imerzoukene, Raphaële Germi, Marion Le Marechal, Aurélie Truffot, Gaëlle Fiard, Bénédicte Janbon, Diane Giovannini, Paolo Malvezzi, Lionel Rostaing, Johan Noble

**Affiliations:** 1 Nephrology, Hemodialysis, Apheresis and Kidney Transplantation Department, Universitary Hospital Grenoble, 38000 Grenoble, France; 2 Medicine Faculty, University of Grenoble Alpes, 38000 Grenoble, France; 3 Virology Department, Universitary Hospital Grenoble, 38000 Grenoble, France; 4 Infectious Disease Department, Universitary Hospital Grenoble, 38000 Grenoble, France; 5 Urology Department, Universitary Hospital Grenoble, 38000 Grenoble, France; 6 Pathology Department, Universitary Hospital Grenoble, 38000 Grenoble, France

**Keywords:** BK virus infection, mTOR inhibitors, renal transplantation

## Abstract

BK virus-associated nephropathy (PvAN) increases the risk of graft failure justifying treatment. Conversion to mammalian target of rapamycin inhibitors (mTORi) and Human polyclonal immunoglobulins (IVIg) could prevent the risk of PvAN. Our retrospective study assessed the efficacy of mTORi associated with IVIg therapy (mTORi±IVIg group) versus standard immunosuppression reduction to clear BKV DNAemia. Among forty-three kidney-transplanted patients with positive BKV DNAemia, we included twenty-six patients in the mTORi±IVIg group and reduced immunosuppression therapy for seventeen patients. We focused on BKV DNAemia clearance on the first-year. Renal function, rejection rate, evolution to PvAN, and complications of immunosuppression were assessed. BKV DNAemia decreased faster and significantly in the control group as compared to the mTORi±IVIg group (*p* < 0.001). Viral clearance was significantly higher in the control group compared to the mTORi±IVIg group (88% vs. 58%; *p* = 0.033). Death-censored graft loss, rejection rates and kidney-graft function at 12 months did not significantly differ. Multivariate analyses significantly associated BKV DNAemia clearance with reducing immunosuppression (OR = 0.11 (0.06–0.9), *p* = 0.045), female kidney donor (OR = 0.10 (0.01–0.59/)], *p* = 0.018) and time to first DNAemia, (OR = 0.88 (0.76–0.96), *p* = 0.019). In our study, the standard treatment for BKV DNAemia had better outcomes than an mTORi±IVIg conversion.

## 1. Introduction

Kidney transplantation (KT) is the optimal therapeutic option for end-stage kidney disease in terms of patient survival, quality of life, and healthcare savings. The most frequent complications associated with immunosuppressive drugs, including calcineurin inhibitors (CNI), are cancers and infections that appear to be correlated with the intensity of the immunosuppression [1]. One of the most common viral infections post-KT is BK virus (BKV) infection. This polyomavirus was first identified in 1971, isolated from the urine of a renal-allograft recipient with ureteric obstruction, but its pathogenicity was initially underestimated [2,3].

Primary BKV infection occurs during childhood, with a worldwide seroprevalence of about 75% among adults. The virus then persists life long, mainly within the reno-urinary tract [4]. Post-KT, the virus may reactivate and multiply within the reno-urinary tract, leading to the destruction of tubular epithelial cells and, subsequently, to a virus-associated nephropathy that can cause chronic graft dysfunction and an increased risk of graft loss [5]. In previous studies, BKV DNAemia was estimated to occur in 11–13% of KT recipients, with 8% having BK/polyomavirus-associated nephropathy (PvAN) [6,7].

Identified risk factors include donor determinants, such as a deceased donor, a female donor, ABO incompatibility, and systemic factors at post-KT (such as acute tubular necrosis and acute rejection, use of corticosteroids, and powerful immunosuppression, especially tacrolimus) [8,9]. A systematic review by Johnston et al. and the 2019 Guidelines of the American Society of Transplantation recommended a reduction in immunosuppression as the first-line treatment for BK DNAemia and PvAN [8,10]. The incidence of graft failure has been reported to be similar following acute cellular rejection and PvAN, which justifies active care of BKV infections. However, lowering immunosuppression increases the risk of rejection [11,12]. Some experimental and clinical studies suggest that mammalian target of rapamycin inhibitors (mTORi) have a specific antiviral effect on BKV tubular epithelial-cell replication, and that conversion from calcineurin inhibitors to mTORi, plus lowering immunosuppression, may prevent the risk of PvAN [13,14]. A mTORi-based regimen is associated with a lower incidence of BKV DNAemia, but the studies failed to prove a benefit to treat an ongoing infection [15]. Another approach to BKV therapy is human IV polyclonal immunoglobulin (IVIg) preparations, which contain BKV-neutralizing antibodies [16]. IVIg administration is associated with an increase in BKV antibody titers in KT recipients, especially for the genotype I BKV (which is the most common) [17]. However, the efficacy of IVIg as a potential treatment for PvAN is controversial [18,19,20,21,22,23]. Overall, the strategy to reduce the tacrolimus level to reduce the risk of BKV added to the potential antiviral effect of mTORi and IVIg may be an interesting approach to treat BKV DNAemia.

To date, no studies have assessed the efficacy of mTORi conversion associated with IVIg to treat BKV infection in KT recipients. In this retrospective study, we assessed the clearance of BKV DNAemia in KT patients treated with IVIg combined with mTORi conversion and low tacrolimus target as compared to the standard of care (i.e., reducing immunosuppression alone).

## 2. Materials and Methods

### 2.1. Study Population

De novo KT recipients are regularly assessed for BKV DNAemia during the first year post-transplantation. In this retrospective single-center study, we looked for all adult KT recipients having had positive blood BKV DNAemia between 3 March 2013 and 7 October 2020. The patients had to meet the following inclusion criteria: conversion to mTORi after the first BKV DNAemia ± adjunction of polyclonal IVIgs in the mTORi±IVIg group; the control group underwent reduced immunosuppression after being tested positive for BKV DNAemia. Patients who were treated with mTORi at the moment of first detection of BKV DNAemia were excluded. All medical data were collected from our database (CNIL (French National committee for data protection) approval number 1987785v0).

### 2.2. BKV Quantification

Positive BKV DNAemia was defined by a positive quantitative polymerase chain reaction (qPCR) of ≥500 copies/mL (=2.69 log10 copies/mL) in the blood. Nucleic acids were extracted from blood using MagNaPure LC^®^ (Roche Diagnostics, Grenoble, France) automatic extraction technology and from urine using EasyMag^®^ (Biomérieux, Marcy Étoile, Lyon, France) automatic extraction technology. The BK viral load was measured by real-time qPCR using a BKV Rgene^®^ kit (Biomérieux, Marcy Étoile, Lyon, France) on a LightCycler^®^ 480 Instrument II (Roche Diagnostics, Grenoble, France). Sustained DNAemia was defined by a PCR > 4 log10 copies/mL (10,000 copies/mL) on two successive tests [24]. BKV DNAemia clearance was defined as a blood qPCR of <500 copies/mL or an undetectable viral load. PvAN was defined by a histologically detected tubulointerstitial inflammation associated with basophilic intrnuclear inclusions in tubular epithelial cells and/or positivity of immunostaining with SimianVirus 40 (Anti-SV40 T-antigen-clone Pab416-Abcam-1:50-Manual technique). Presumptive PvAN was defined as plasma BKV DNAemia > 10,000 copies/ mL (4 log10 copies/mL) [8]. Posttransplantation monitoring of BKV in recipients was done in the center every three months during the first year and then in the case of clinical events such as rejection or increase of creatininemia without an identified cause, according to the KDIGO guidelines. In the case of positive BKV DNAemia: the viremia is controlled at two weeks, and then monitored monthly until clearance.

### 2.3. Endpoints

The primary endpoint was BKV DNAemia clearance during the first year post-therapeutic intervention. The DNAemia kinetics were collected from the medical intervention until the 12th month and beyond. We also assessed the estimated glomerular filtration rate (eGFR) at one year, graft loss, death, evolution to PvAN (proven by non-systematic biopsies), and treatment tolerability. Antibody-mediated rejection and cellular rejection were biopsy-proven according to the most recent updated Banff classification at the time of the biopsy. Biopsies were systematically performed at 3 months post-KT and then for cause thereafter. eGFR was calculated using the CKD-EPI method and was expressed in mL/min/1.73 m^2^. Treatment intolerability was considered when treatment was discontinued due to its side effects.

### 2.4. Immunosuppression

Immunosuppression at post-KT consisted of a triple therapy: mycophenolate mofetil (MMF, 1 g/day), tacrolimus (with trough levels targeted at between 6 and 8 ng/mL after 1 month post-KT), and prednisolone (given at 500 mg pre-operatively, then progressively tapered to 10 mg/day, and then discontinued at month 3 in the absence of rejection or a donor-specific antibody, but was maintained for IgA nephropathy patients).

The date of medical intervention was defined by the day that treatment was modified, i.e., conversion to mTORi±IVIg or reduced immunosuppression.

In the mTORi±IVIg group, mTORi (everolimus) was introduced to obtain a trough level of between 5 and 7 ng/mL, MMF was discontinued, and the tacrolimus dose was reduced to reach trough levels of between 3 and 5 ng/mL. IVIg were given at a minimal dose of 1.2 g/kg (0.2 g/kg × 6 or 0.4 g/kg × 3.

In the control group, reduced immunosuppression included reduction or discontinuation of corticosteroids and/or MMF and/or tacrolimus.

Data on tacrolimus trough levels were collected for up to one year before medical intervention and at six months after to assess the impact of over or lower immunosuppression on BKV clearance.

### 2.5. Statistical Analyses

Symmetrically and heterogeneous quantitative variables are shown as means ± standard deviations, and median (ranges), respectively. Qualitative variables are expressed as percentages. Student’s *t*-test or the Mann–Whitney U test was used to compare continuous variables. A linear regression model was used to compare the BKV DNAemia kinetics over time between the two groups. The chi-square test or Fisher’s exact test was used to compare qualitative variables. Variables with a significance of *p* < 0.05 in univariate analyses were included in the multivariate analyses. We used a multivariate regression model to predict BKV DNAemia clearance. In the multivariate analyses, we included all significant parameters associated with BKV DNAemia clearance in our model and we included the use of Rituximab at transplantation because of the potential bias. A *p*-value of <0.05 was considered statistically significant. Analyses were performed using R software.

## 3. Results

### 3.1. Cohort Characteristics

#### 3.1.1. Clinical Characteristics

**Figure 1:**
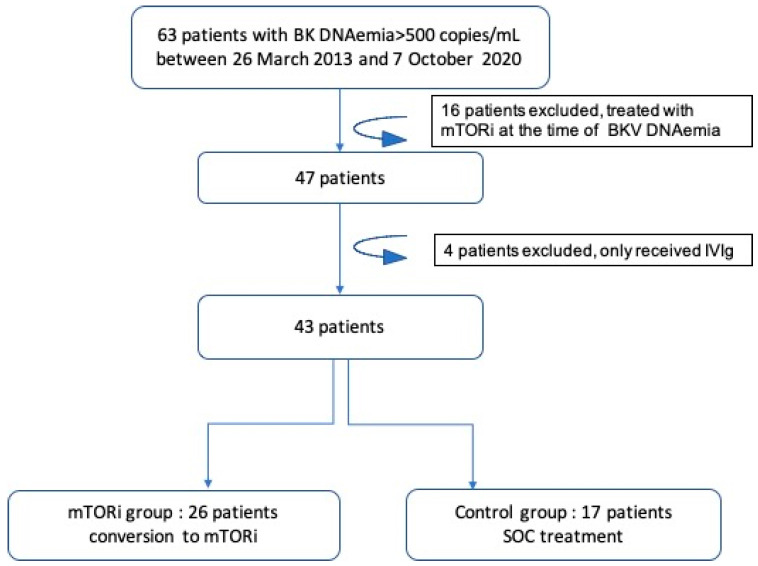
Flowchart of patients. mTORi: mammalian target of rapamycin inhibitor; IVIg: intravenous immunoglobulins; SOC: standard-of-care treatment.

- Between 26 March 2013 and 7 October 2020, 67 patients had a positive BKV blood DNAemia of >500 copies/mL (22%). Forty-seven patients did not have an mTORi-based regimen at the time of the first incidence of DNAemia. Among these, 26 patients were converted to mTORi with or without simultaneous infusion of IVIgs (mTORi±IVIg group) and 17 underwent reduced immunosuppression (control group, i.e., standard of care). Eighteen patients (69%) received IVIgs as well as mTOR. The t//Total IVIg mean dose was 94.7 ± 60 g (1.3 g/kg).
- Ten patients did not meet the inclusion criteria (see lowchart in Figure 1). Baseline characteristics of the included population are shown in Table 1. Clinical characteristics, such as age at time of DNAemia, gender, body-mass index (BMI), initial nephropathy, diabetes mellitus pre-KT, and vascular impairment, were similar between the two groups. The mTORi±IVIg group was more likely to have received a graft from a living donor, a younger donor, an ABO-incompatible donor and to receive Rituximab after KT as compared to the control group.
- Median follow-up time of the study population from medical intervention until the last follow-up was 45 (25.7–70.5) months: with a shorter time for the mTORi±IVIg group compared to the control group (respectively, 32.3 (23.8–47.7)/] vs. 77.3 (64.6–91) months, *p* < 0.001).

#### 3.1.2. BKV at the Time of Medical Intervention

- BKV DNAemia at the time of medical intervention did not significantly differ between the mTORi±IVIg group and the control group: i.e., respectively, 3.8 ± 0.9 log10 copies/mL vs. 4.2 ± 0.6 log10 copies/mL, *p* = 0.15. Biopsy-proven PvAN (SV40+) at the time of medical intervention did not significantly differ between the two groups: ten patients (38%) in the mTORi±IVIg group versus one patient (6%) in the control group (*p* = 0.28).

### 3.2. Primary Endpoint and BKV DNAemia Outcomes

**Figure 2:**
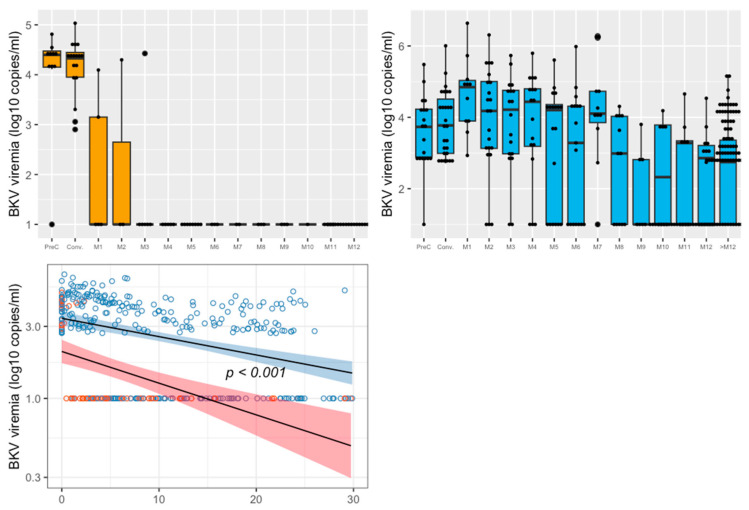
Upper figures: BKV DNAemia (log10 copies/mL) at the time of medical intervention, each month from M1 to M12 and after 1 year post-treatment in both groups. The control group (immunosuppression reduction) is represented in the upper-left panel and the mTORi±IVIg group in the upper-right panel. BKV: BK virus; mTORi: mammalian target of rapamycin inhibitor, PreC: pre-conversion, i.e., before medical intervention. Conv: conversion, i.e., medical intervention. Bottom figure: BKV DNAemia kinetics (log10 copies/mL) over time between medical intervention and M12 in both groups. The mTORi±IVIg group is represented in blue, the control group is represented in red. Undetectable BKV DNAemia was considered as 1 log10/mL to allow graphical representation.

- The median times between KT and BKV DNAemia when medical intervention was conducted were similar: i.e., 3.6 (2.8–7.1) months for the mTORi±IVIg group, and 3.6 (3.0–4.6) months for the control group (*p* = 0.84).
- Regarding the primary endpoint, the prevalence of BKV DNAemia clearance during the first year post-medical intervention was significantly higher in the control group compared to the mTORi±IVIg group (88% vs. 58%; *p* = 0.03).
- Figure 2 represents the kinetics of BKV blood DNAemia over time for both groups. Using a linear regression model, we showed that the decreasing slopes of BKV DNAemia over time differed significantly between the mTORi±IVIg and control groups, in favor of the control group (*p* < 0.001).
- At month 12 post-medical intervention, the viral load was 2.3 ± 1.3 log10 copies/mL in the mTORi±IVIg group, whereas no patient had positive BKV DNAemia in the control group (*p* = 0.10). We then assessed, for each month, the BKV DNAemia level in blood after the medical intervention (Figure 2). In the control group, BKV decreased rapidly at month 3 to then remain negative for beyond one year. Conversely, in the mTORi±IVIg group, BKV DNAemia decreased more slowly, and many more patients still hadll positive viral loads after one year. Mean BKV DNAemia in the mTORi±IVIg group was 3.8 (1.4) log10/mL at month 3, at 2.9 (1.8) log10/mL at month 6, at 2.3 (1.3) log10/mL at month 12, and 2.3 (1.4) log10/mL beyond the first year after medical intervention.
- During the follow-up, three patients (11%) developed biopsy-proven PvAN: all were in the mTORi±IVIg group.

### 3.3. Kidney Outcomes

#### 3.3.1. Graft Survival

**Figure 3:**
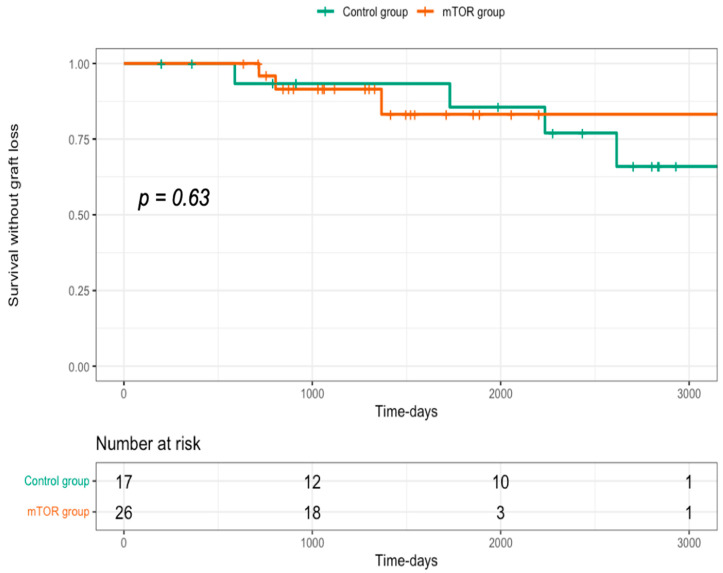
Outcomes of death-censored graft loss (DCGL) according to BKV DNAemia treatment. Kaplan–Meier analysis curve, showing death-censored graft survival rates for patients who received mTORi for BKV DNAemia compared to the control group.

The prevalence of death-censored graft loss (DCGL) did not differ between the mTORi±IVIg and control groups: 15% and 23% in the mTOR and control group, respectively (*p* = 0.50). This represented an absolute risk reduction of 8% (number to treat = 12.5) for the benefit of the control group. DCGL occurred after a median time of 45.1 (25.7–70.5) months after medical intervention, i.e., 32.3 (23.8–47.7) months for the mTORi±IVIg group and 77.3 (64.5–91) months for the control group (*p* = 0.63). Figure 3 shows the Kaplan–Meyer survival curves of DCGL in both groups. Among the causes of graft loss, PvAN was incriminated in three patients in the mTORi±IVIg group and chronic active rejections occurred in four patients from the control group. No patient lost their graft during the first year post-medical intervention.

#### 3.3.2. Rejection Rate after the Medical Intervention

Twenty-two patients (85%) had a biopsy post intervention in the mTORi±IVIg group and 12 patients (70%) in the control group. The rate of T-cell-mediated rejection at the end of follow-up did not differ statistically significantly between the mTORi±IVIg and control groups: 11% versus 18%, respectively (*p* = 0.74). The percentages of biopsy-proven antibody-mediated rejections were similar between the mTORi±IVIg and control groups: 15% versus 23%, respectively, *p* = 0.68. During the period of follow-up, no patients developed de novo DSA post medical intervention.

#### 3.3.3. Kidney Function

- At the time of medical intervention, mean eGFR was 56 ± 19 mL/min/1.73 m^2^. There was no statistical difference in eGFR between the groups: 58.5 ± 19 mL/min/1.73 m^2^ in the mTORi±IVIg group and 52.8 ± 18.2 mL/min/1.73 m^2^ in the control group (*p* = 0.325). Similarly, the median proteinuria level at the time of medical intervention was 0.3 [0.1–0.3] g/L. Proteinuria also did not statistically differ between the two groups: 0.3 [0.2–0.3] g/L versus 0.2 [0–0.3] g/L in the mTORi±IVIg group and control groups, respectively (*p* = 0.09).
- Kidney-graft function at 12 months post-medical intervention did not significantly differ between the mTORi±IVIg and control groups, respectively: 54.3 ± 19.4 mL/min/1.73 m^2^ and 51.5 ± 24.4 mL/min/1.73 m^2^ (*p* = 0.468). Proteinuria at 12 months post-medical intervention was significantly higher in the mTORi±IVIg group compared to the control group: 0.3 [0.2–0.8] g/L versus 0.1 [0–0.2] g/L, respectively, *p* = 0.004.

### 3.4. Immunosuppression Management and Tolerability

We then assessed the potential influence of the dose of immunosuppression on BKV outcomes. Patients in the mTORi±IVIg group received the same mean ATG-induction dose as the control group: 4.6 mg/kg, versus 3.8 mg/kg (*p* = 0.37).

During the first year before medical intervention, 317 tacrolimus trough concentrations were assessed. The median tacrolimus trough level was 7.7 [5.8–9.5] ng/mL in the mTORi±IVIg group and 7.5 [5.8–10.2] ng/mL in the control group (*p* = 0.89). Tacroilimus and everolimus were maintained for all patients during the first year of follow-up in the mTORi±IVIg group even in the case of persisting BKV DNAemia.

In the control group, reduction of immunosuppression consisted of the various protocols summarized in Table 2. Regarding mTORi tolerability, 85% of patients in the mTORi±IVIg group continued everolimus at the end of follow-up.

Opportunistic infection rates during the first year did not significantly differ between the mTORi and control groups: respectively, 11% versus 11% of infections (including infection-induced hospitalization and CMV infections), *p* = 0.98. The development of cancer did not significantly differ between the mTORi and control groups: respectively, 11% versus 23% (*p* = 0.29).

### 3.5. Factors Associated with BKV DNAemia Clearance

**Figure 4:**
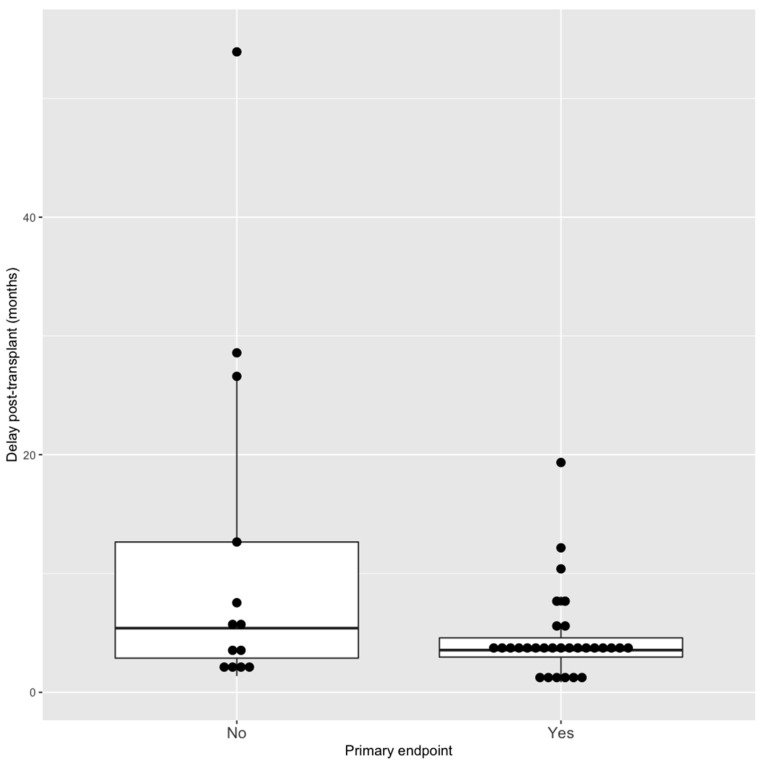
Comparison of the occurrence of BKV DNAemia post transplantation regarding to the primary endpoint (BKV clearance). Delay of viremia is reported in months. Boxplots are separated between those who did not reach the primary endpoint: “No” and those who cleared BKV DNAemia: “Yes”.

- We also assessed factors associated significantly with our primary endpoint: i.e., BKV DNAemia clearance at 1 year after medical intervention (Table 3).
- In univariate analysis, receiving a kidney from a female donor and reduced immunosuppression versus mTORi conversion were significantly associated with BKV DNAemia clearance. Time of occurrence between transplantation and medical intervention did not significantly differ between the BKV DNAemia clearance groups: 3.6 [3.0–4.6] months versus 5.4 [2.9–12.7] months for persistent BKV DNAemia patients (*p* = 0.14), nor did lymphocyte level at the time of medical intervention. The use of IVig was not associated to BKV DNAemia clearance in univariate analysis neither in a model when associated with mTOR conversion (OR = 0.33 [0.04–1.9], *p* = 0.24).
- In the multivariate model, we included all significant factors associated with BKV clearance and we included the delay of BKV occurrence post transplantation because the association was really close to significance and a shorter delay of initial viremia appeared to be significantly associated with BKV endpoint. Three factors remained significantly associated with less BKV DNAemia clearance at 1 year in this model: mTORi conversion (vs. reducing immunosuppression), having a male donor (as defined in Section 2) and a shorter delay of BKV DNAemia occurrence post transplantation (Figure 4): OR = 0.11 [0.06–0.9] *p* = 0.045 and 0.10 [0.01–0.59] *p* = 0.018 and 0.88 [0.76–0.96] *p* = 0.019, respectively.

## 4. Discussion

We retrospectively compared two therapeutic strategies for BKV infection. The prevalence of BKV DNAemia clearance at 1 year was greater in KT recipients who had reduced immunosuppression compared to those who were converted to mTORi. Moreover, in patients still viremic at 1 year, BKV viral load was significantly higher in the mTORi±IVIg group. The other factors significantly associated with less BKV DNA clearance in our study were having a male donor and a shorter delay of BKV DNAemia occurrence post transplantation. To the best of our knowledge, those factors were never found be associated with BKV evolution post kidney transplantation in the literature.

Most published studies have compared the effect of mTORi on BKV DNAemia prevention but not on BKV DNAemia treatment.

Reduced immunosuppression is the first recommended step to manage BKV infection [8]. Reducing immunosuppression has shown efficacy in large prospective studies, although no randomized controlled trials are currently ongoing. One long-term study investigated outcomes at 6 years post-transplantation following a reduced immunosuppression protocol to treat sustained BKV DNAemia. Graft survival, clinical rejection, and eGFR were similar to those of non-viremic KT patients [25].

Successful clearance of BKV-positive DNAemia and the prevention of PvAN after KT relies on the efficiency of the immune system and, more precisely, specific T-cell immunity [26]. A review published in 2017 by Ambalathingal et al. reports that BKV latency and reactivation are likely dependent on a stable antiviral memory T-cell response and humoral passive immunity [27].

There is in vitro evidence for the antiviral activity of mTORi. Comparison of the effects of mTORi and tacrolimus on BKV replication in primary human renal-tubular epithelial cells [13] showed that sirolimus impaired BKV replication, with rapid and effective inhibition during early viral gene expression, but not during later viral gene expression. This is consistent with current data that report a lower incidence of BKV infection in patients already treated with mTORi and may explain why mTORi efficacy on active BKV infections has not yet been proven. mTORi, like sirolimus, does not inhibit BKV-specific T-cell activation [28], nor does it impair cytokine secretion and cytotoxic capacity of BKV-specific T cells [29]. Moreover, mTOR inhibition promotes memory-cell generation in mice, and may improve the response of virally induced memory T cells [30].

The use of mTORi was associated with a reduced incidence of BKV DNAemia in the TRANSFORM study compared to standard exposure to tacrolimus [31]. Similarly, the OPTN American registry analysis showed a significantly lower incidence of BKV infection in transplant recipients treated with mTORi compared to other immunosuppressive treatments [32]. However, a meta-analysis of randomized controlled trials did not highlight a reduction in BKV infections in patients receiving mTORi therapy [33].

A small study compared KT recipients of whom seven were treated with mTORi for an active BKV infection, eight were undergoing reduced immunosuppression, and fifteen patients were receiving cidofovir and IVIgs. Improvement in BKV clearance in plasma and urine was shown in the mTORi group [34] but the reduced immunosuppression and cidofovir groups received several lines of treatment, making it difficult to interpret those results. Moreover, a prospective randomized study with 40 patients compared conversion to an everolimus strategy versus a 50% reduction in MMF to treat BKV infection after KT and showed no statistically significant difference in BKV DNAemia clearance at 3 months [15], which agrees with our results.

Passive humoral immunity appears to be more effective during the early phases of viral replication. This may explain why we could not show a clear benefit of IVIgs to treat active established BKV infections. In 2010, it was shown that human IVIgs contain BKV neutralizing antibodies that can cause in vitro inhibition of viral DNA. The effect was lower when treatment was delayed after anti-viral inoculation [16]. More recently, a team from Strasbourg Hospital (France) reported that IVIgs may be a valuable preventive strategy for BKV replication. They confirmed that IVIgs administration increased neutralizing antibody titers in KT recipients [17]. They also found that the incidence of BKV DNAemia in a high-risk group (defined by a low rate of neutralizing antibodies) was similar to that in a low-risk group (with an initial high rate of neutralizing antibodies) after supplementing the high-risk group with IVIgs [24].

A series of case reports with poor levels of proof reported the efficacy of IVIgs as a treatment for active BKV infection. Sener et al. treated eight patients with biopsy-proven PvAN by reducing immunosuppression and giving IVIgs. In half of their patients, BKV DNAemia was negative after 15 months of follow-up [18]. Histological resolution of PvAN and a reduction in BKV DNAemia was described after IVIg therapy was given to pediatric KT recipients with persistent BKV DNAemia and after immunosuppression was reduced and/or cidofovir therapy [19,35]. In 2020, Matsumura et al. treated five cases of PvAN with IVIgs: three cases had decreased blood BKV DNAemia, and large SV40 T antigens became reduced on all graft biopsies [22].

Some studies with larger cohorts have been published, although they have often used combined immunosuppressive strategies. Vu et al. reported in 2015 on clearing viremia by 90% in 30 patients treated with IVIgs after unsuccessfully lowering immunosuppression and leflunomide therapy [20]. A recent retrospective study assessed the benefit of IVIg therapy. Eighty-six patients with proven BK virus nephropathy were separated into an intervention group receiving IVIg therapy and a control group treated with leflunomide and ciprofloxacin. Forty-seven of the 52 (90%) IVIg-treated patients had lowered BKV DNA loads of <500 copies/mL [23].

A retrospective, single-center cohort study observed more BKV clearance and a faster decrease in BKV DNAemia in a group that received IVIg as well as cyclosporine, leflunomide, ciprofloxacin, and intravenous cidofovir [36].

In our study, the presence of a control group that received the recommended standard of care, with no other treatment modifications, may explain this difference. We may argue that reducing immunosuppression alone could have played the same role as IVIgs in these patients.

This study has some limitations because of its small cohort size and its retrospective design. Our two groups were formed from a historical cohort of patients treated between 2013 and 2020, which included time-related changes to the standard of care. The mTORi±IVIg group included more ABO-incompatible KTs who had not undergone the same initial immunosuppression regimen (more patients received Rituximab post transplantation) and were at more risk for BKV infection, as suggest by Schachtner et al. [37]. However, except the age and donor type, the two groups were broadly similar, particularly regarding the initial BKV viral load. We may suppose that Rituximab influenced viral clearance in the mTOR group and may explain the poorer outcome in this group on the primary endpoint. This potentially highlights the deleterious role of rituximab for BKV clearance and should be considered in future studies. Another limit of the study was the heterogenicity of the immunosuppressive regimen in the control group. The choice was made to include all types of immunosuppressive regimen reduction in order to avoid a selection bias due to the severity of BKV.

Due to the retrospective design of our study, immunosuppression protocols were not formally codified. Indeed, delays in medical intervention after the first positive BKV DNAemia were heterogeneous depending on the practitioner, despite a common initial protocol. In the same way, reduced immunosuppression in the control group was not standardized, but was left to the discretion of the physician. However, these variabilities in strategies of care for BKV infections reflect the current common practice and, thus, real-life situations.

## 5. Conclusions

Our study showed that the actual standard of care, i.e., reduction of immunosuppression alone to treat BKV DNAemia post KT as compared to a protocol with mTORi conversion ± IVIg seems to be more effective as a treatment of BKV DNAemia but with a possible higher risk of T-cellmediated rejection and death-censored graft loss. We also showed that BKV DNAemia clearance was associated with male donor and with a shorter delay of DNAemia occurrence post KT. The finding should be further investigated in larger randomized prospective studies.

## Figures and Tables

**Table 1:** Baseline characteristics of the population.

	mTORi±IVIg Group *n* = 26	Control Group *n* = 17	Total *n* = 43	*p*-Value
Age (years)	56.3 (16.3)	60.5 (10.4)	58 (14.3)	0.502
Male *n* (%)	22 (85)	16 (94)	38 (88)	0.342
BMI (kg/m^2^)	25.8 (3.5)	26.1 (5.4)	25.9 (4.3)	0.691
Diabetes *n* (%)	3 (11)	2 (12)	5 (12)	0.982
High blood pressure *n* (%)	23 (88)	15 (88)	38 (88)	0.982
Nephropathy *n* (%)				0.556
- Diabetic	1 (4)	2 (12)	3 (7)	
- Nephroangiosclerosis	2 (8)	3 (18)	5 (12)	
- Glomerulopathy	9 (35)	6 (35)	15 (30)	
- Other	14 (54)	6 (35)	20 (46)	
Vascular impairment *n* (%)				
- Arteriopathy	0	1 (6)	1 (5)	0.429
- Stroke	0	0	0	0.211
Gender of donor: female *n* (%)	16 (61)	11 (65)	27 (63)	0.834
Type of donor *n* (%)				<0.001
- NHBD	5 (19)	1 (6)	6 (14)	
- Encephalic death	6 (23)	15 (88)	21 (49)	
- Living donor	15 (58)	1 (6)	16 (37)	
Age of donor (years)	53.2 (16.9)	63.9 (11.4)	57.5 (15.7)	**0.037**
ABO-incompatible *n* (%)	4 (15)	0	4 (9)	0.141
HLA-incompatible *n* (%)	4 (15)	0	4 (9)	0.141
Transplantation rank				0.238
1	21 (81%)	11 (65%)	32 (74)	
2	5 (19%)	6 (35%)	11 (25)	
ATG *n* (%)	22 (85)	100	39 (91)	0.140
Mismatch I *n* (%)				0.764
[0–2]	11 (42)	6 (35)	17 (39)	
[3, 4]	14 (54)	11 (65)	25 (58)	
Mismatch II *n* (%)				0.373
[0–2]	12 (46)	13 (76)	25 (58)	
[3, 4]	13 (50)	4 (23)	17 (39)	
Rituximab *n* (%)	8 (31)	0	8 (19)	**0.014**
Immunosuppression at medical intervention				
- MMF *n* (%)	25 (96)	100	42 (98)	0.413
- MMF mean dose mg/day	952.3 (250)	1062.5 (403)	994.3 (317)	0.192
Corticoids *n* (%)	25 (96)	15 (88)	40 (93)	0.151
- Corticoids mean dose mg/day	7 (7.9)	5.7 (4.2)	6.5 (6.8)	0.796
- Tacrolimus *n* (%)	100%	100%	100%	
- Tacrolimus median dose mg/day	6.6 [5.7–8.9]	7.2 [5.4,10.1]		0.825
Lymphocytes G/L	0.7 [0.4–1]	0.6 [0.5–0.9]		0.902

ABO: blood group; ATG: Antithymoglobulin; BMI: Body Mass Index; HLA: Human leukocyte antigen; IVIg: Intravenous immunoglobulins; MMF: mycophenolate mofetil; mTORi: mammalian target of Rapamycin inhibitors; NHBD: non heart beating donor. Bold *p*-values are considered significant (*p* < 0.05).

**Table 2:** Protocols for reducing immunosuppression in the control group.

Immunosuppression Modifications in the Control Group	*n*
Lowering tacrolimus (trough level 3–5 ng/mL)	5
Lowering MMF (500 mg/day)	1
Lowering MMF (500 mg/day) and tacrolimus (trough level 3–5 ng/mL) *	4
Lowering MMF (500 mg/day) and tacrolimus and stopping steroids *	1
Stopping steroids	3
Stop steroids and lower tacrolimus (trough level 3–5 ng/mL) *	2
Stop MMF and lower tacrolimus (trough level 3–5 ng/mL) *	1
Total	17

* Modifications done within a period of 1 month. MMF: mycophenolate mofetil

**Table 3:** Univariate and multivariate analyses of factors associated with BKV DNAemia clearance at 1 year after treatment.

	Univariate Analysis		Multivariate Analysis	
	Odd-Ratio [2.5–97.5%]	*p*-Value	Odd-Ratio [2.5–97.5%]	*p*-Value
Recipient gender: male	4.2 [0.61–35.63]	0.145	-	-
Recipient age	0.97 [0.92–1.02]	0.307	-	-
Donor gender: male	0.22 [0.05–0.87]	0.035	**0.10 [0.01–0.59]**	**0.02**
Living donor		0.179	-	-
Rituximab use	0.34 [0.07–1.73]	0.188	0.36 [0.04–2.7]	0.33
Donor age	1.01 [0.96–1.05]	0.937	-	-
ATG dose	1.00 [0.99–1.01]	0.321	-	-
Tac trough concentration at medical intervention	1.14 [0.86–1.57]	0.486	-	-
IVIg use	0.79 [0.44–1.18]	0.495	-	-
High lymphocyte number at medical intervention	3.59 [0.62–29.40]	0.182	-	-
eGFR (mL/min/1.73 m^2^) at medical intervention	1.00 [0.96–1.04]	0.676	-	-
Delay between KT and BKV	0.90 [0.78–0.98]	0.066	**0.88 [0.76–0.96]**	**0.019**
mTORi versus reduced immunosuppression	0.10 [0.01–0.48]	0.033	**0.11 [0.06–0.9]**	**0.04**

ATG: Antithymoglobulin; eGFR: estimated Glomerular Filtration Rate; IVIg: Intravenous immunoglobulins; mTORi: mammalan target of Rapamcin inhibitors. Bold results are considered significant (*p* < 0.05).

## Data Availability

The data presented in this study are available on request from the corresponding author.
